# Exploring the Nexus Between Alcohol Consumption, Sleep Deprivation, and Memory Among Male Students at a Tertiary Institution in Ghana

**DOI:** 10.1002/brb3.70744

**Published:** 2025-08-04

**Authors:** Daniel L. Egbenya, Lord B. Amponsah, Ireneaus Nyame, El‐Gibbor C. Rolinklaka, Rose Nanabin, Lovinger M. Nyave, Stephen B. G. Oppong, Jephthah K. N. Boadi, Dzifianu A. Edoh‐Torgah, Isaac B. Mensah, Akua A. Karikari

**Affiliations:** ^1^ Department of Physiology, School of Medical Sciences, College of Health and Allied Sciences University of Cape Coast Cape Coast Ghana; ^2^ Department of Biomedical Sciences, School of Allied Health Sciences, College of Health and Allied Sciences University of Cape Coast Cape Coast Ghana

## Abstract

**Background:**

Alcohol consumption and poor sleep quality may affect cognitive function and overall health in young adults. The rising trend of alcohol use among university students, particularly males, coupled with the prevalence of sleep deprivation, poses a substantial risk for cognitive decline. This study aimed to evaluate the association between alcohol consumption, sleep deprivation, and cognitive function among male university students.

**Methods:**

A cross‐sectional survey was conducted among 242 male students at a university in Ghana. The Alcohol Use Disorder Identification Test (AUDIT), Pittsburgh Sleep Quality Index (PSQI), and neuropsychological tests such as the Rey Auditory Verbal Learning Test (RAVLT), Trail Making Test (TMT A and B), and the International HIV Dementia Scale were used to assess alcohol prevalence, sleep quality, and memory function, respectively.

**Results:**

Alcohol prevalence stood at 52.5%, along with poor sleep quality at 61.2% among participants. Alcohol use risk levels and sleep quality scores were significantly positively correlated (*r* = 0.29, *p* = 0.001). Additionally, PSQI subscales, sleep disturbance (*p* = 0.001) and daytime dysfunction (*p* = 0.001), were significantly positively correlated with AUDIT scores. Risky alcohol consumers with poor sleep quality status showed outcomes indicative of possible cognitive decline at statistically significant levels and were four times more likely to be classified as having a possible cognitive deficit (*p* = 0.038, AOR = 4.4, 95% CI = 1.09–17.70) compared to a no alcohol consumption with good sleep status group.

**Conclusion:**

The outcome of this study shows that alcohol usage and poor sleep quality among male university students are associated with cognitive deficit; hence, the need for targeted interventions.

## Graphical abstract

Among male Ghanaian university students, alcohol consumption and poor sleep quality are highly prevalent and positively correlated. Both factors are associated with cognitive and memory deficits, highlighting the need for targeted health education and interventions.

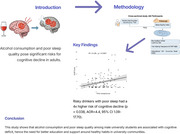

## 1 Introduction

Alcohol consumption and poor sleep quality are significant risk factors for cognitive decline in adults (Köhler et al. 2023; Kohler and Hofmann 2015). Globally, alcohol consumption has steadily increased among young adults, and it is the leading risk factor for many diseases and mortalities, contributing to approximately 3 million (5.3%) deaths annually and 132.6 million (5.1%) disability‐adjusted life years (DALYs) (GBD 2016 Alcohol and Drug Use Collaborators 2018). Approximately 13.5% of all mortality cases in young people aged 20–39 years are attributed to excessive alcohol consumption (Park and Kim 2020), with a prevailing trend among university students (Hingson et al. 2009). Due to the unique social, psychological, and academic challenges that confront university students, alcohol consumption and poor sleep quality may become a common lifestyle and occurrence (Lorant et al. 2013). Some studies in Africa have identified poor sleep quality and growing tendencies in alcohol consumption predominantly among male tertiary students on the continent (GBD 2016 Alcohol and Drug Use Collaborators 2018; Aderinto et al. 2024; Ajayi et al. 2019; Deressa and Azazh 2011; Gebreslassie et al. 2013; Boltana et al. 2023). A high prevalence of poor sleep quality, which negatively impacts academic performance, has been described among tertiary students from Ghana, Ethiopia, and Nigeria, independent of their alcohol status (Aderinto et al. 2024; Lankrew Ayalew et al. 2022; Simpong et al. 2025). On alcohol consumption, data from Ghana, Nigeria, South Africa, and Ethiopia revealed that females are less likely to consume alcohol as compared to males (Ajayi et al. 2019; Boltana et al. 2023; Kyei‐Gyamfi et al. 2023; Govender et al. 2017). This gender disparity in alcohol consumption could be an indication of the imminent social stigma directed at women who drink, which is more pronounced in certain cultures, especially in the African setting (Sudhinaraset et al. 2016). Moreover, females are typically viewed in many societies as the guardians of culture and social norms, and alcohol abuse in women often provokes social outrage (Ajayi et al. 2019; Wilsnack et al. 2005). One of the harmful effects of chronic alcohol use is brain impairment and associated cognitive decline (Nunes et al. 2019). For instance, alcohol intake has been linked to significant cognitive deficits, including impairments in attention and memory, which may result in decreased academic performance and higher dropout rates (Kenney et al. 2014). Alcohol consumption and poor sleep quality can interact to produce a synergistic effect that further exacerbates cognitive impairment (Sirtoli et al. 2022). Alcohol has been shown to disrupt sleep architecture, particularly by suppressing rapid eye movement (REM) sleep, which is crucial for cognitive processes such as memory consolidation, integration, and emotional regulation (Park et al. 2015). Also, alcohol's impact on neurotransmitter systems, particularly its facilitation of γ‐aminobutyric acid (GABA) function, can lead to initial sedation but ultimately results in fragmented sleep patterns that impair cognitive performance (Lohsoonthorn et al. 2013; Thakkar et al. 2015). The disruption of sleep homeostasis can create a cycle where poor sleep leads to increased alcohol consumption as individuals seek to self‐medicate their sleep disturbances, further compounding cognitive issues (Helaakoski et al. 2022).

Very few studies have been conducted on either the combined effects of alcohol intake and sleep deprivation among university students in sub‐Saharan Africa, particularly in Ghana. Such studies are essential to inform policymakers about the need to enforce policies to regulate the harmful use of alcohol amongst students and to augment support services to students. In the present study, we sought to investigate the association among alcohol intake, sleep deprivation, and memory in male students at the University of Cape Coast in Ghana. To the best of our knowledge, the present work is the first in Ghana to assess the associations between alcohol consumption, sleep deficiency, and cognitive performance in a student population. Male students were chosen for this study because alcohol consumption in Ghana is largely dominated by men (Boachie et al. 2022). In addition, women who consume alcohol are generally stigmatized (Griffin et al. 2020), which would impact the participation of female students in this work. Specifically, we first determined the prevalence of alcohol use and sleep quality among the general male student population. We subsequently isolated persons who consumed alcohol along with their sleep quality status and further explored their associations with memory performance.

## 2 Materials and Methods

### 2.1 Study Design

The study employed an institution‐based cross‐sectional research design utilizing quantitative data to assess the variables of interest (alcohol consumption, sleep quality, and cognitive decline) at the University of Cape Coast, a public university located in Cape Coast, the Central region of Ghana. A questionnaire and various cognitive tests were administered to the participants. Male undergraduate and postgraduate students attending the University of Cape Coast were included in the study. The students were recruited from four colleges on the university campus, including the College of Health and Allied Sciences (CoHAS), the College of Humanities and Legal Studies (CHLS), the College of Agriculture and Natural Sciences (CANS), and the College of Education Studies (CES). The fifth college, the College of Distance Education (CoDE), was excluded from the study because students in this college are largely off campus.

### 2.2 Sample Size and Sampling Technique

The sampling technique used in recruiting participants for the study was a simple random sampling. Data collection booths were mounted at the various sites on campus, and male students were invited randomly by the researchers to participate in the study. The Cochran formula (*n*₀ = (*z* ^2^ × *p* × (1 − *p*))/*e* ^2^) with a confidence interval set at 95% and a margin of error at 5% (0.05) (*n*₀ = (1.96^2^ × 0.5 × (1 − 0.5))/0.05^2^ = 384) was used. After correcting for a finite population of about 16533, *n* = *n*₀ / [1 + (*n*₀ − 1) / *N*], where *n*₀ = 384, *N* = 16533; and *n* = 384 / [1 + (384 − 1)/16533] = 375 (UoCC 2023). After adjusting for a potential nonresponse rate of 10%, a sample size of 417 was obtained. A final sample size of 242 was used due to time and resource constraints and unforeseen nonresponse from some participants. Additionally, four participants, who had either a neurological condition or were on psychotropic medication, were excluded from any further analysis in line with the study's exclusion criteria. All participants signed informed consent forms before participating in the study. The researchers ensured the tests were conducted in a quiet and conducive environment. Tests were filled in person, and administration took approximately 25 min per participant.

### 2.3 Data Collection

The self‐structured questionnaire consisted of three main sections: demographic data such as age, year of study, college of study, alcohol consumption (frequency, quantity, and type of alcohol consumed), and sleep patterns (sleep duration, sleep quality, and sleep disorders). Data on potential confounding variables were obtained by collecting information on whether participants had any neurological condition, including mental health disorders, and the participants’ use of medications known to impair cognitive function.

#### 2.3.1 Alcohol Use Identification

The Alcohol Use Disorders Identification Test (AUDIT) was used to measure alcohol consumption risk levels among alcohol consumers. The 10‐item AUDIT questionnaire was chosen for the current study to provide a standardized and objective measure of the levels of alcohol consumption among the participants. Scores on the AUDIT range from 0 to 40, with higher scores signifying a greater potential of health and safety risks associated with alcohol use. Participants who consumed alcohol were categorized as low‐risk (0–7), risky (8–12), and high‐risk (13+) based on AUDIT scores (Reinert and Allen 2002). Varying levels of alcohol consumption patterns were observed as recorded by the AUDIT, resulting in the categorization of participants as low risk, risky, and high risk based on their consumption patterns (units consumed) and a host of other factors as recorded by the AUDIT.

#### 2.3.2 Sleep Quality Measures

Also, the English language version of the Pittsburgh Sleep Quality Index (PSQI) for measuring sleep quality was used to gather information on sleep efficiency and other critical sleep quality parameters (Buysse et al. 1989). Participants completed the 19‐item PSQI. Each of the items belongs to one of seven component scores: sleep duration, sleep, sleep disturbance, use of sleeping medication, daytime dysfunction due to sleepiness, sleep efficiency, and overall sleep quality. Scores for each question ranged from 0 (*no difficulty*) to 3 (*severe difficulty*). The component scores were subsequently added up to achieve a global PSQI score, and a score > 5 revealed participants with poor sleep quality (Buysse et al. 1989).

#### 2.3.3 Cognition and Memory Assessment

Furthermore, we used the International HIV Dementia Scale (IHDS) to assess the cognition and memory of participants due to its ease of use, simplicity, and reliability (Dang et al. 2015). The administration of the dementia scale involved three tasks: memory recall, finger tapping, and psychomotor speed. The overall dementia scale score, ranging from 0 to 12, assesses the likelihood of possible cognitive decline (dementia), with scores less than 10 an indication of a higher risk of dementia. While the IHDS is primarily used for HIV/AIDS populations, it can be used to assess cognitive decline among non‐HIV/AIDS study populations as well. For instance, the IHDS was used to assess cognitive deficits in subjects with neurological diseases associated with subcortical damage and human African trypanosomiasis (Montanucci et al. 2021; Njamnshi et al. 2020).

For the evaluation of verbal learning and memory, the Rey Auditory Verbal Learning Test (RAVLT) was conducted. Participants were tested as per the prescriptions and instructions of the original RAVLT (Rey 1958). Different summary scores were derived from the raw RAVLT scores. A point was scored for each correct word, giving a total of 15 per round of recall. There were seven rounds of recall in total (Tests 1–5, Test B, and Test 6). Five different summary scores were derived from the raw RAVLT scores. The sum of the first five recalls was grouped into the Immediate Total Recall score (< 30 = Poor; ≥ 30 = Good). The result of the difference between the scores for Tests 1 and 5 constituted the Learning score (≤ 0 = Poor; > 0 = Good) (Teruya et al. 2009). Finally, we also performed the trail making test (TMT), where TMT A measures visual scanning, attention, and psychomotor speed, while TMT B tests for higher executive function and working memory (Du et al. 2022). The cutoff for deficiency in TMT A was > 78 s, and that of TMT B was > 273 s (Ciolek and Lee 2020). Participants were grouped based on their AUDIT risk levels and sleep quality status, and a logistic regression analysis was performed against their neuropsychological outcomes. Data collection was done from June to September 2024.

### 2.4 Inclusion and Exclusion Criteria

The study included only male undergraduate and postgraduate students attending the University of Cape Coast. Female students and students from the CoDE were excluded from this study. Other exclusion criteria were male undergraduate and postgraduate students of the University of Cape Coast who had an underlying mental or neurological condition or were on any medication relating to the management of cognitive disorders. Prospective participants with underlying mental or neurological conditions or who were on any medication relating to the management of cognitive disorders were included in the data collection but excluded from data analysis to prevent them from harboring any feeling of being stigmatized which might lead them to possibly give misleading information (with its attendant problems for the study's validity).

### 2.5 Statistical Analysis

Data collected from participants was processed, cleaned, and validated using Google Sheets and exported to IBM Statistical Package for Social Sciences (SPSS) version 27 for coding and statistical analysis. The data from the questionnaire included demographic information about the participants. The Fisher's Exact test was used to ascertain the relationships between the demographics and variables of interest (i.e., sleep quality and alcohol consumption prevalence) due to cell counts less than five. Pearson's correlation analysis was performed among alcohol consumers to identify the relationship between global PSQI scores and AUDIT scores. A binary logistic regression was performed to predict the cognitive outcomes of classified groups based on their AUDIT risk levels and sleep quality. Statistical significance was set at a two‐sided alpha level of 0.05 and *p* values less than 0.05 were considered statistically significant.

### 2.6 Ethical Approval

The Institutional Review Board at the University of Cape Coast, Ghana, reviewed and approved this study (ethical clearance ID: UCCIRB/CHAS/2024/109). Participation of the students in the study was voluntary, and written informed consent was obtained from each participant before data collection. It is worth mentioning that in Ghana, the legal age for granting nonsexual consent is 12 years and above. The youngest age recorded was 17 years; thus, they could give their consent for the study (Ghartey 2023). Study participants were informed about the anonymity and confidentiality of the questionnaires. The names of the students were not recorded on the questionnaire. Instead, their preferred initials were used. During the data analysis process, participants were de‐identified, and codes were assigned instead. Measures were also taken to ensure the respect and dignity of all study participants, with the freedom to withdraw from the study at any point in time, if they so wish, clearly stated.

## 3 Results

### 3.1 Socio‐Demographic Characteristics of Participants

Out of the 242 male university students, the majority (81.0%) were aged between 21 and 25 years. The lowest number of participants (1.7% each) were those less than 18 years and those between 30 and 33 years old. Most of the students (36.4%) were from the CHLS, followed by 24.0% from the CoHAS. More than a third of the participants (36.0%) were level 400 students, and 5.8% were postgraduate students (Table 1).

**TABLE 1:** Socio‐demographics of Study Participants.

Socio‐demographic characteristics	Frequency	Percentage (%)
Age		
<18	4	1.7
18–20	13	5.4
21–25	196	81.0
26–29	25	10.3
30–33	4	1.7
Level of Study		
Undergraduate		
100	43	17.8
200	52	21.5
300	46	19.0
400	87	36.0
Postgraduate		
500	8	3.3
600	5	2.1
700	1	0.4
College		
CANS	49	20.2
CHLS	88	36.4
CES	46	19.0
CoHAS	59	24.4

Abbreviations: CANS, College of Agriculture and Natural Sciences; CES, College of Education Studies; CHLS, College of Humanities and Legal Studies; CoHAS, College of Health and Allied Sciences.

### 3.2 Alcohol Consumption and Sleep Quality Prevalence

Alcohol prevalence among 242 participants stood at 52.5%, representing 127 male students who consumed alcohol. Using the PSQI global scores, participants with poor and good sleep quality constituted 61.2% and 38.8%, respectively. To determine the alcohol use risk levels of participants, we used the AUDIT scores of participants to categorize them as low‐risk consumers (83.5%), risky consumers (11.0%), and high‐risk consumers (5.5%), as shown in Table 2.

**TABLE 2:** Prevalence of alcohol consumption and sleep quality.

Variables	Frequency	Percentage (%)
Alcohol Consumption Prevalence	*n* = 242	
No	115	47.5
Yes	127	52.5
Sleep Quality	*n* = 242	
Poor	148	61.2
Good	94	38.8
Alcohol Use Risk Level	*n* = 127	
Low risk	106	83.5
Risky	14	11.0
High risk	7	5.5

#### 3.2.1 Relationship Between Participants’ Demographic Information and Alcohol Consumption and Sleep Quality

Age was found to be significantly associated with alcohol consumption (*p* = 0.003). Among students aged between 21 and 25, 49.5% consumed alcohol, while 50.5% did not. The level of study was found to be significantly associated with alcohol consumption (*p* = 0.005), with 60.9% of third‐year students and 57.5% of fourth‐year students consuming alcohol. Age was significantly associated with PSQI sleep quality status (*p* = 0.043). Among students aged between 21 and 25, 64.3% were classified as poor sleepers, while 35.7% had good sleep quality status. Level of study was significantly associated with PSQI sleep quality status (*p* = 0.017), with 71.1% of second‐year students and 63.2% of fourth‐year students classified as poor sleepers, as shown in Table 3.

**TABLE 3:** Bivariate analysis between demographics and prevalence of alcohol consumption and sleep quality.

Demographics	Alcohol prevalence		Fisher's exact *p* value	Sleep quality		Fisher's exact *p* value
	No *n* (%)	Yes *n* (%)		Good *n* (%)	Poor *n* (%)	
Age			**0.003**			**0.043**
<18	3 (75.0)	1 (25.0)		1 (25.0)	3 (75.0)	
18–20	8 (61.5)	5 (38.5)		6 (46.2)	7 (53.8)	
21–25	99 (50.5)	97 (49.5)		70 (35.7)	126 (64.3)	
26–29	4 (16.0)	21 (84.0)		13 (52.0)	12 (48.0)	
30–33	1 (25.0)	3 (75.0)		4 (100.0)	0 (0.0)	
Level of study			**0.005**			**0.017**
Undergraduate						
100	30 (69.8)	13 (30.2)		23 (53.5)	20 (46.5)	
200	27 (51.9)	25 (48.1)		15 (28.9)	37 (71.1)	
300	19 (39.1)	28 (60.9)		14 (30.4)	32 (69.6)	
400	37 (42.5)	50 (57.5)		32 (36.8)	55 (63.2)	
Postgraduate						
500	3 (37.5)	5 (62.5)		5 (62.5)	3 (37.5)	
600	0 (0.0)	5 (100.0)		4 (80.0)	1 (20.0)	
700	0 (0.0)	1 (100.0)		1 (100.0)	0 (0.0)	
College of study			0.354			0.275
CANS	26 (53.1)	23 (46.9)		21 (42.9)	28 (57.1)	
CHLS	36 (40.9)	52 (59.1)		39 (44.3)	49 (55.7)	
CES	21 (45.7)	25 (54.3)		13 (28.3)	33 (71.7)	
CoHAS	32 (54.2)	27 (45.8)		21 (35.6)	38 (64.4)	

Abbreviations: CANS, College of Agriculture and Natural Sciences; CES, College of Education Studies; CHLS, College of Humanities and Legal Studies; CoHAS, College of Health and Allied Sciences. *p* < 0.05.

### 3.3 Relationship Between Alcohol Consumption and Sleep Quality

**FIGURE 1:**
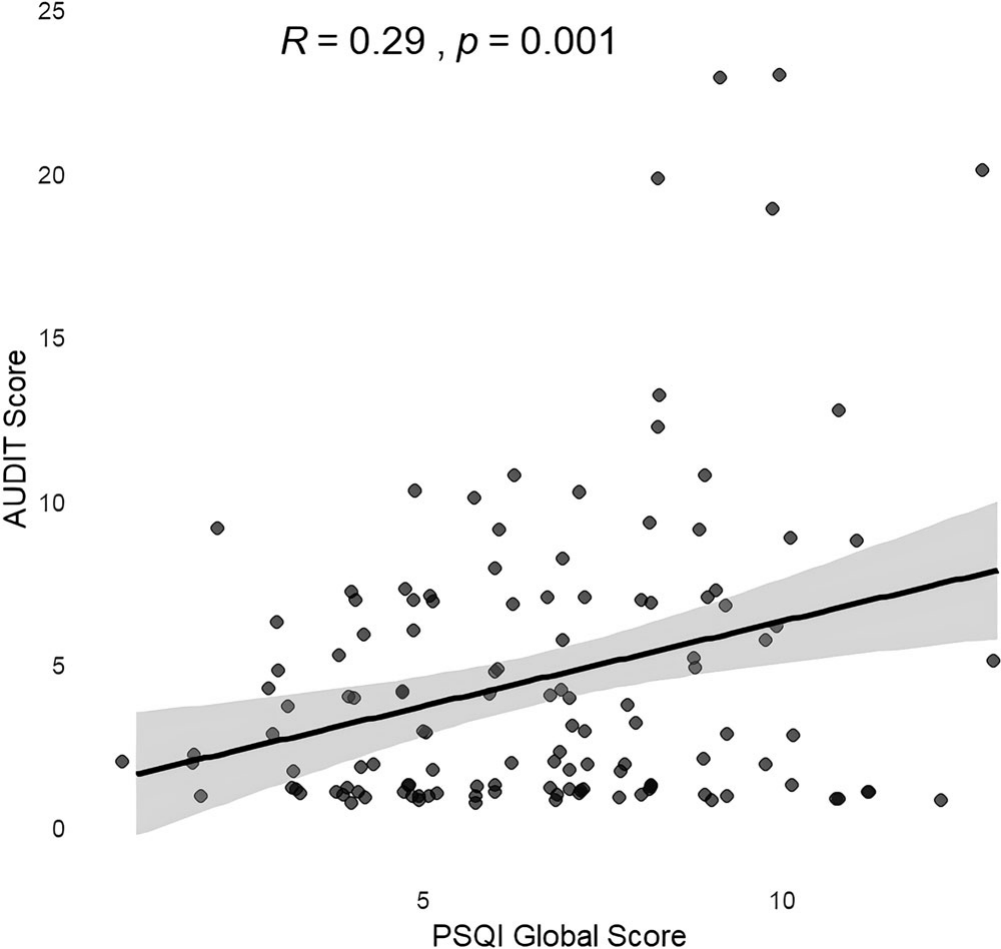
Correlation between AUDIT and PSQI scores.

Correlation analysis performed among alcohol consumers showed a significant weak to moderate positive linear correlation (*r* = 0.29, *p* = 0.001) between AUDIT scores and PSQI scores. This implies that higher AUDIT scores correlated with higher global PSQI scores (Figure 1).

Figure 1. Bivariate Pearson's correlations of AUDIT and PSQI scores. PSQI and AUDIT scores were significantly positively correlated (*r* = 0.29, *p* = 0.001).

### 3.4 Correlations Between PSQI Components and AUDIT Scores

Pearson's correlation was conducted between the global PSQI component scores and AUDIT scores among alcohol consumers. Sleep Disturbances (*r* = 0.39, *p* = 0.001) and Daytime Dysfunction (*r* = 0.34, *p* = 0.001) showed a moderate positive correlation with AUDIT scores, such that more sleep disturbances and difficulty in maintaining wakefulness were correlated with greater AUDIT scores. Meanwhile, parameters such as sleep latency and use of sleep medications showed no significant relationship with AUDIT scores (Table 4).

**TABLE 4:** Relationship between subscales of PSQI and AUDIT scores.

PSQI components	AUDIT scores	
	*p* value	*r*
Subjective Sleep Quality	0.449	0.07
Sleep Latency	0.221	0.11
Sleep Duration	0.162	0.13
Habitual Sleep Efficiency	0.733	−0.03
Sleep Disturbances	**0.001**	**0.39**
Use of Sleep Medications	0.527	0.06
Daytime Dysfunction	**0.001**	**0.34**

*p* < 0.05.

### 3.5 Interaction Between Alcohol Use Risk Level and Sleep Quality on Memory and Cognition

Participants were further classified into seven groups based on their alcohol use disorder risk levels and their sleep quality: G1 (no alcohol—good sleep), G2 (no alcohol—poor sleep), G3 (low‐risk—good sleep), G4 (low‐risk—poor sleep), G5 (risky—poor sleep), G6 (risky—good sleep), and G7 (high‐risk—poor sleep). After performing a binary logistic analysis, no significant relationship was found between any of the groups and learning status and immediate recall memory (Tables 5A and 5B). For dementia status assessment, G5 (risky–poor sleep) participants showed signs of possible neurocognitive impairments and were four times more likely to be classified as having neurocognitive deficits (G5: *p* = 0.038, AOR = 4.4, 95% CI = 1.09–17.70) compared to G1 (no alcohol‐good sleep) (Table 5C). In TMT A, G7 (high‐risk‐poor sleep) participants also showed impairments (Table 5D), whilst in TMT B, alcohol risk groups G4 and G7 showed no deficiency (Table 5E).

**TABLE 5A:** Association between short‐term memory and AUDIT‐PSQI groups.

Groups	RAVLT Learning Status		*p* value	COR (95% CI)	*p* value	AOR (95% CI)
	Good *n* (%)	Poor *n* (%)				
G 1	48 (100.0)	0 (0.0)	—	1		
G 2	68 (98.5)	1 (1.5)	1.000	0.99 (0.00–99)	0.998	2.17e^7^ (0.00–99)
G 3	43 (100)	0 (0.0)	1.000	1.00 (0.00–99)	1.000	1.27 (0.00–99)
G 4	59 (96.7)	2 (3.3)	0.998	5.40e^6 (0.00–99)	0.998	5.33e^7^ (0.00–99)
G 5	11 (100.0)	0 (0.0)	1.000	1.00 (0.00–99)	1.000	1.21 (0.00–99)
G 6	3 (100.0)	0 (0.0)	1.000	1.00 (0.00)	1.000	1.36 (0.00–99)
G 7	7 (100.0)	0 (0.0)	1.000	1.000	1.000	0.98 (0.00–99)

Abbreviations: AOR, adjusted odds ratio; CI, confidence interval; COR, crude odds ratio; RAVLT, Rey Auditory Verbal Learning Test.

**TABLE 5B:** Association between immediate memory AUDIT‐PSQI groups.

Groups	RAVLT Immediate Recall Status		*p* value	COR (95% CI)	*p* value	AOR (95% CI)
	Good *n* (%)	Poor *n* (%)				
G 1	43 (89.5)	5 (10.5)	—	—		
G 2	69 (100.0)	0 (0.0)	0.997	0.00 (0.00–99)	0.997	0.00 (0.00–99)
G 3	43 (100.0)	0 (0.0)	0.998	0.00 (0.00–99)	0.997	0.00 (0.00–99)
G 4	61 (98.3)	1 (1.7)	0.997	0.00 (0.00–99)	0.997	0.00 (0.00–99)
G 5	10 (91)	1 (9.0)	0.896	0.86 (0.09–8.19)	1.000	1.00 (1.01–9.9)
G 6	3 (100.0)	0 (0.0)	0.999	0.00 (0.00–99)	0.999	0.00 (0.00–99)
G 7	7 (100.0)	0 (0.0)	0.999	0.00 (0.00–99)	0.999	0.00 (0.00–99)

Abbreviations: AOR, adjusted odds ratio; CI, confidence interval. COR, crude odds ratio; RAVLT, Rey Auditory Verbal Learning Test.

**TABLE 5C:** Association between cognition and AUDIT‐PSQI groups.

IHDS						
Groups	Dementia Status		*p* value	COR (95% CI)	*p* value	AOR (95% CI)
	Normal *n* (%)	Possible Dementia *n* (%)				
G 1	34 (70.8)	14 (29.2)	—	1	—	1
G 2	45 (65.2)	24 (34.8)	0.524	1.3 (0.58–2.87)	0.486	1.30 (0.59–2.97)
G 3	32 (74.4)	11 (25.6)	0.702	0.8 (0.33–2.11)	0.608	0.80 (0.29–2.03)
G 4	40 (65.6)	21 (34.4)	0.560	1.3 (0.56–2.88)	0.513	1.30 (0.58–2.99)
G 5	4 (36.4)	7 (63.6)	0.039	4.3 (1.07–16.85)	**0.038**	**4.40 (1.09–17.70)**
G 6	1 (33.3)	2 (66.7)	0.212	4.9 (0.41–57.9)	0.219	4.80 (0.39–58.76)
G 7	3 (42.9)	4 (57.1)	0.155	3.2 (0.64–16.38)	0.140	3.40 (0.67–17.33)

Abbreviations: AOR, adjusted odds ratio; CI, confidence interval; COR, crude odds ratio; IHDS, International HIV Dementia Scale. *p* < 0.05.

**TABLE 5D:** Association between psychomotor skills (TMT A) and AUDIT‐PSQI groups.

Groups	TMT A Deficient		*p* value	COR (95% CI)	*p* value	AOR (95% CI)
	Yes *n* (%)	No *n* (%)				
G 1	1 (2.1)	47 (97.9)	—	1	—	1
G 2	4 (5.8)	65 (94.2)	0.349	0.35 (0.04–3.19)	0.240	0.25 (0.03–2.52)
G 3	6 (14.0)	37 (86.0)	0.065	0.13 (0.15–1.14)	0.140	0.18 (0.02–1.7)
G 4	6 (9.8)	55 (90.2)	0.137	0.20 (0.23–1.68)	0.120	0.17 (0.18–1.59)
G 5	2 (18.2)	9 (81.8)	0.066	0.10 (0.01–1.17)	0.086	0.10 (0.01–1.37)
G 6	0 (0.0)	3 (100.0)	0.999	3.40e^6^ (0.00–99)	0.999	1.60e^7^ (0.00–99)
G 7	3 (42.9)	4 (57.1)	**0.005**	0.03 (0.00–0.34)	**0.005**	0.26 (0.00–0.33)

Abbreviations: AOR, Adjusted Odds Ratio; CI, Confidence Interval; COR, Crude Odds Ratio; TMT, Trail Making Test. *p* < 0.05.

**TABLE 5E:** Association between psychomotor skills (TMT B) and AUDIT‐PSQI groups.

Groups	TMT B Deficient		*p* value	COR (95%CI)	*p* value	AOR (95%CI)
	Yes *n* (%)	No *n* (%)				
G 1	0 (0.0)	48 (100)	—	1		1
G 2	1 (1.4)	68 (98.6)	0.998	0.00 (0.00–99)	0.998	0.00 (0.00–99)
G 3	2 (4.7)	41 (95.3)	0.997	0.00 (0.00–99)	0.997	0.00 (0.00–99)
G 4	0 (0.0)	61 (100.0)	1.000	1.00 (0.00–99)	1.000	0.91 (0.00–99)
G 5	2 (18.2)	9 (81.8)	0.997	0.00 (0.00–99)	0.997	0.00 (0.00–99)
G 6	1 (33.3)	2 (66.7)	0.997	0.00 (0.00–99)	0.997	0.00 (0.00–99)
G 7	0 (0.0)	7 (100.0)	1.000	1.00 (0.00–99)	1.000	0.84 (0.00–99)

Abbreviations: AOR, adjusted odds ratio; CI, confidence interval; COR, crude odds ratio; TMT, trail making test.

## 4 Discussion

The associations between alcohol consumption, sleep quality, cognition, and memory among male students at the University of Cape Coast in Ghana were determined in this research. We also assessed the prevalence of alcohol consumption and sleep deprivation among the participants. Most of our participants were within the 21–25‐year age group. This corresponds to the age bracket of university students when one takes into consideration the school‐going age in the country (Lawer Egbenya and Kwesi Quayson 2022).

### 4.1 Alcohol Consumption Prevalence

Alcohol prevalence in this study was found to be 52.5% among the male students examined. Previous studies in Ghana, Nigeria, China, Ireland, and the United Kingdom highlighted that male students consume more alcoholic beverages than females (Ajayi et al. 2019; Kyei‐Gyamfi et al. 2023; Wang et al. 2023; Davoren et al. 2016). Though this work did not involve female students, our data revealed a higher prevalence of alcohol consumption than studies by Aboagye et al. (2021), which reported a prevalence of 47% among male students in Ghana. This may be suggestive of a growing pattern of alcohol use on Ghanaian campuses. Additionally, we showed that students belonging to the 21–25 age group consumed more alcohol compared to students of the other age groups. This is consistent with findings from Aboagye et al. (2021), who discovered a higher prevalence of alcohol consumption among this age bracket. This age group may consume alcohol as a coping mechanism, or they may be influenced by the curiosity to indulge in alcohol intake as a way to display their adulthood (Aboagye et al. 2021). More fourth‐year students consumed alcohol compared to first‐year students. With increasing academic and social demands of university education as students progress to higher levels, they are faced with peer influence and other psychosocial factors that impact their decision to consume alcohol to cope with these factors (Aboagye et al. 2021), which could account for the above observation.

### 4.2 Sleep Quality

Using the global PSQI scores to identify sleep quality, a significant proportion of participants (61.2%), especially those in their fourth year, were categorized as having poor sleep quality. This implies that many tertiary students experience sleep difficulties and aligns with previous research by Lund et al. (2010), who also reported that over 60% of the students who participated in their investigations experienced poor sleep quality. In Ghana, the prevalence of poor sleep among tertiary students, as reported by Simpong et al. (2025) and Yeboah et al. (2022), stood at 48.5% and 54.1%, respectively, which are lower than the prevalence observed in our study. Some previous studies attribute poor sleep quality in tertiary students to academic pressures and individual lifestyle issues (Hershner and Chervin 2014; Orzech et al. 2016), which could account for the variation in prevalence reported among university students in Ghana. Poor sleep quality is associated with decreased academic performance, impaired memory, and increased risk of mental health issues among university students (Gaultney 2010), and the combination of sleep deprivation and alcohol consumption can have a synergistic effect on cognitive impairment (Kenney et al. 2012).

### 4.3 Alcohol and Sleep Interaction

Furthermore, our analysis using Pearson's correlation revealed a significantly weak to moderate positive correlation between AUDIT and global PSQI scores among alcohol consumers, indicating that higher alcohol use correlates with poor sleep quality. The AUDIT scores also correlated with sleep disturbances and daytime dysfunction among the participants. Chronic alcohol intake has been proven to affect sleep quality by reducing REM sleep, which results in prolonged sleep disturbances over time, creating a physiologic disruption in sleep homeostasis (Koob and Colrain 2020). This could create a bidirectional relationship between these two factors, with alcohol consumption initiating a cycle of sleep deprivation and poor sleep quality, precipitating a habit of alcohol drinking (Helaakoski et al. 2022). Current knowledge from sleep studies shows that alcohol use disorders can particularly influence sleep disruptions and wakefulness even among young people and may be underpinned by changes to circadian rhythm and sleep architecture (Dzierzewski et al. 2022).

### 4.4 Alcohol and Sleep Association With Memory Performance

Our data showed a positive association between risky alcohol consumers who also experienced poor sleep quality and their psychomotor skills and memory scores, resulting in their categorization as likely dementia candidates as per the IHDS classification. This positive association indicates that alcohol consumption and poor sleep quality may be linked with dementia risk levels even among a youthful population. Heavy alcohol drinking is associated with an increased risk of dementia (Jeon et al. 2023). Interestingly, we observed that some individuals who did not consume alcohol and reported having good sleep quality (G1) showed notable possible dementia status. This may be attributable to other factors such as hypertension, obesity, and other psychosocial challenges that were not captured in this research. Studies have shown that a notable prevalence of hypertension and obesity exists among university students in Ghana, Nigeria, and Ethiopia (Gyamfi et al. 2018; Kadiri 2024; Tadesse and Alemu 2014). In the TMT A test, which evaluates visuomotor skills and processing speed, the high‐risk alcohol consumers with poor sleep group (G7) showed some deficiencies, which implies a possible effect of alcohol on visual scanning, attention, and psychomotor speed. For the TMT B, which tests for higher executive function and working memory, there were no significant deficiencies observed in the various groups. This is contrary to a study that showed poor TMT B performance among alcohol consumers (Day et al. 2013). It is worthy to note that performance in the TMT B test correlates with the level of education of participants (Płotek et al. 2014). As such, it was demonstrated by Souza et al. (2013) that patients with Parkinson's disease who had more education performed better in the TMT B test than their counterparts who were less educated. These results suggest that academic activities may provide some neuroprotective effects and improve the cognitive reserve among young males even in the midst of risk factors like alcohol consumption (Wilson et al. 2019).

### 4.5 Limitations

The study's cross‐sectional nature does not provide evidence for elucidating causal relationships and/or effects between alcohol consumption, sleep deprivation, and memory deficits. The study's focus on only male students limits the generalizability of its findings to university students in general. Future studies could include female students to investigate differences in alcohol and sleep quality prevalence, as well as their overall associations with memory task performances compared to their male counterparts.

## 5 Conclusion

This study provides evidence of the association between alcohol consumption, sleep deprivation, and cognitive function among male university students. The findings indicate a significant prevalence of both alcohol use and poor sleep quality among the study participants, which correlated with notable impairments in cognitive performance, particularly in areas such as memory. This research suggests that the interplay between alcohol consumption and sleep deprivation exacerbates cognitive deficits, further indicating a synergistic correlation that warrants further investigation. Specifically, the data revealed that higher levels of alcohol intake were associated with poorer sleep quality, which in turn is negatively associated with cognitive abilities. This relationship underscores the importance of addressing both factors concurrently in interventions aimed at improving the cognitive health of university students.

## Author Contributions

**Daniel L. Egbenya**: writing – original draft, methodology, software, formal analysis, writing – review and editing, supervision, validation. **Lord B. Amponsah**: investigation, writing – original draft, methodology, formal analysis. **Ireneaus Nyame**: investigation, methodology, software, formal analysis. **El‐Gibbor C. Rolinklaka**: investigation, methodology, software, writing – original draft. **Rose Nanabin**: investigation, methodology, formal analysis, writing – original draft. **Lovinger M. Nyave**: investigation, methodology, software, formal analysis. **Stephen B.G. Oppong**: investigation, methodology, formal analysis. **Jephthah K.N. Boadi**: investigation, formal analysis, methodology. **Dzifianu A. Edoh‐Torgah**: investigation, methodology, formal analysis. **Isaac B. Mensah**: investigation, methodology, software, validation. **Akua A. Karikari**: conceptualization, writing – original draft, writing – review and editing, validation, supervision, project administration, methodology, formal analysis.

## Conflicts of Interest

The authors declare no conflicts of interest.

## Peer Review

The peer review history for this article is available at https://publons.com/publon/10.1002/brb3.70744

## Data Availability

The data that support the findings of this study are available from the corresponding author upon reasonable request.
